# Transcriptomic analysis of candidate osmoregulatory genes in the eastern oyster *Crassostrea virginica*

**DOI:** 10.1186/1471-2164-15-503

**Published:** 2014-06-20

**Authors:** Laura E Eierman, Matthew P Hare

**Affiliations:** Department of Natural Resources, Cornell University, 213 Bradfield Hall, Ithaca, NY 14853 USA

**Keywords:** *Crassostrea virginica*, Osmoregulation, Transcriptome, dN/dS, Gene enrichment, SNP, Cd-hit

## Abstract

**Background:**

The eastern oyster, *Crassostrea virginica*, is a euryhaline species that can thrive across a wide range of salinities (5-35). As with all estuarine species, individual oysters must be able to regulate their osmotic balance in response to constant temporal variation in salinity. At the population level, recurrent viability selection may be an additional mechanism shaping adaptive osmoregulatory phenotypes at the margins of oyster salinity tolerance. To identify candidate genes for osmoregulation, we sequenced, assembled, and annotated the transcriptome of wild juvenile eastern oysters from ‘high’ and ‘low’ salinity regimes. Annotations and candidates were mostly based on the Pacific oyster (*Crassostrea gigas*) genome sequence so osmoregulatory relevance in *C. virginica* was explored by testing functional enrichment of genes showing spatially discrete patterns of expression and by quantifying coding sequence divergence.

**Results:**

The assembly of sequence reads and permissive clustering of potentially oversplit alleles resulted in 98,729 reftigs (contigs and singletons). Of these, 50,736 were annotated with 9,307 belonging to a set of candidate osmoregulatory genes identified from the *C. gigas* genome. A total of 218,777 SNPs (0.0185 SNPs/bp) were identified in annotated reftigs of *C. virginica*. Amino acid divergence between translations of *C. virginica* annotated reftigs and *C. gigas* coding sequence averaged 23.2 % with an average dN/dS ratio of 0.074, suggesting purifying selection on protein sequences. The high and low salinity source oysters each expressed a subset of genes unique to that group, and the functions for these annotated genes were consistent with known molecular mechanisms for osmotic regulation in molluscs.

**Conclusions:**

Most of the osmoregulatory gene candidates experimentally identified in *C. gigas* are present in this *C. virginica* transcriptome. In general these congeners show coding sequence divergence too high to make the *C. gigas* genome a useful reference for *C. virginica* bioinformatics. However, strong purifying selection is characteristic of the osmoregulatory candidates so functional annotations are likely to correspond. An initial examination of *C. virginica* presence/absence expression patterns across the salinity gradient in a single estuary suggests that many of these candidates have expression patterns that co-vary with salinity, consistent with osmoregulatory function in *C. virginica*.

**Electronic supplementary material:**

The online version of this article (doi:10.1186/1471-2164-15-503) contains supplementary material, which is available to authorized users.

## Background

The eastern oyster (*Crassostrea virginica*) builds reefs that support productive estuarine communities and provide important ecosystem services [1, 2]. However, overfishing, disease pressure, and environmental stress have led to the loss of approximately 90% of biomass across the eastern oyster’s home range since the early 1900’s [3–5]. Two important topics in oyster biology and restoration are the mechanisms by which oysters respond to stress [6, 7] and the ability of oyster populations to either acclimate to stress through phenotypic plasticity or adapt via selection. The majority of work on eastern oysters has focused on immune response to pathogens [8–11] with a few observational studies on other environmental stressors [6, 12]. Spatial and temporal variation in salinity is a given for estuaries, and phenotypic buffering of cell volume through osmolyte control is an essential adaptation for all organisms that live there.

Eastern oysters are found along salinity gradients ranging from near freshwater conditions (salinity of 5) to oceanic salinities (salinity of 35) [13–15]. Their greatest abundance is typically at intermediate salinities, with the adult physiological optimum posited to be as narrow as salinities of 15–18 [15]. At the margins of this environmental envelope, recent results suggest that post-settlement viability selection is one important process for sustaining adult populations [16]. While the genes involved in osmoregulation have not been well characterized in the eastern oyster, recent studies on the Pacific oyster (*Crassostrea gigas*) [17–19] provide valuable tools for investigating the genetics of osmoregulation. Generating genome-scale resources such as transcriptome sequences for *C. virginica* can facilitate studies of gene expression and the physiology of osmoregulation in order to better understand responses to osmotic stress at the individual and population levels.

Oysters regulate cell volume in response to changing salinity through multiple mechanisms. Oysters are osmoconformers with no ability to osmoregulate their extracellular fluid [20]. Salinity fluctuations therefore result in energetically costly processes to maintain isoosmostic balance by accumulating or releasing osmotically active solutes (osmolytes) [20]. These osmolytes include both inorganic ions such as N^+^, K^+^ and Ca^2+^ and organic substances such as free amino acids (FAA) and quaternary amines [21]. Oysters, like many organisms when under great osmotic stress, primarily use organic osmolytes such as taurine, alanine, aspartic acid, glycine, and betaine [21–23]. Organic osmolytes are able to provide osmotic bulk under high osmotic stress without the direct physiological trade-offs that inorganic ions would have [24]. Furthermore, organic osmolytes can stabilize proteins and protect cells from oxidative stress [20, 24]. A variety of functional classes of enzymes are likely involved in osmoregulation, including peptidases to catalyze the hydrolysis of peptides into amino acids, kinases to phosphorylate plasma membrane proteins, and transporters to move molecules across cell membranes [20].

Most molecular physiological studies of osmoregulation in oysters have focused on the products of single genes, such as taurine transporter [25, 26]. The *C. gigas* genome sequence and the initial evaluation of gene expression between salinity treatments [17] demonstrated differential expression for hundreds of genes. Genomic studies of *C. virginica* gene expression across natural salinity gradients have also shown many genes responding to this environmental gradient [6]. To enable more focused future studies on osmoregulation in *C. virginica*, a first step is the identification of candidate genes involved in this core physiological process.

Our objective was to identify genes putatively involved in osmoregulation in the eastern oyster by sequencing, assembling, and annotating the transcriptome from low- and high-salinity source populations of juvenile oysters by using 454 sequencing technology. Using annotations and differential expression data from *C. gigas,* we identified *C. virginica* transcripts that are candidates for osmoregulatory function. Given that these congeners shared a common ancestor more than 82 Mya [27], we explored the functional appropriateness of these annotations in two ways. First, we quantified the distribution of coding sequence divergence and estimated the strength of purifying selection maintaining similar polypeptide sequences in the two species. Second, we tested for predicted expression patterns in normalized cDNA libraries from low- and high-salinity wild oysters. Specifically, we predicted that transcripts found in one salinity population but not the other would be enriched for candidate osmoregulatory genes and for osmoregulation-related gene ontology terms (GO; http://www.geneontology.org). Our evaluation of this transcriptome and results of these associated analyses provide some confidence that these candidate genes are a comprehensive starting point for experiments investigating the physiological and evolutionary responses of eastern oysters to osmoregulatory challenges in their estuarine environment.

## Methods

### Sample collection and archiving

**Figure 1:**
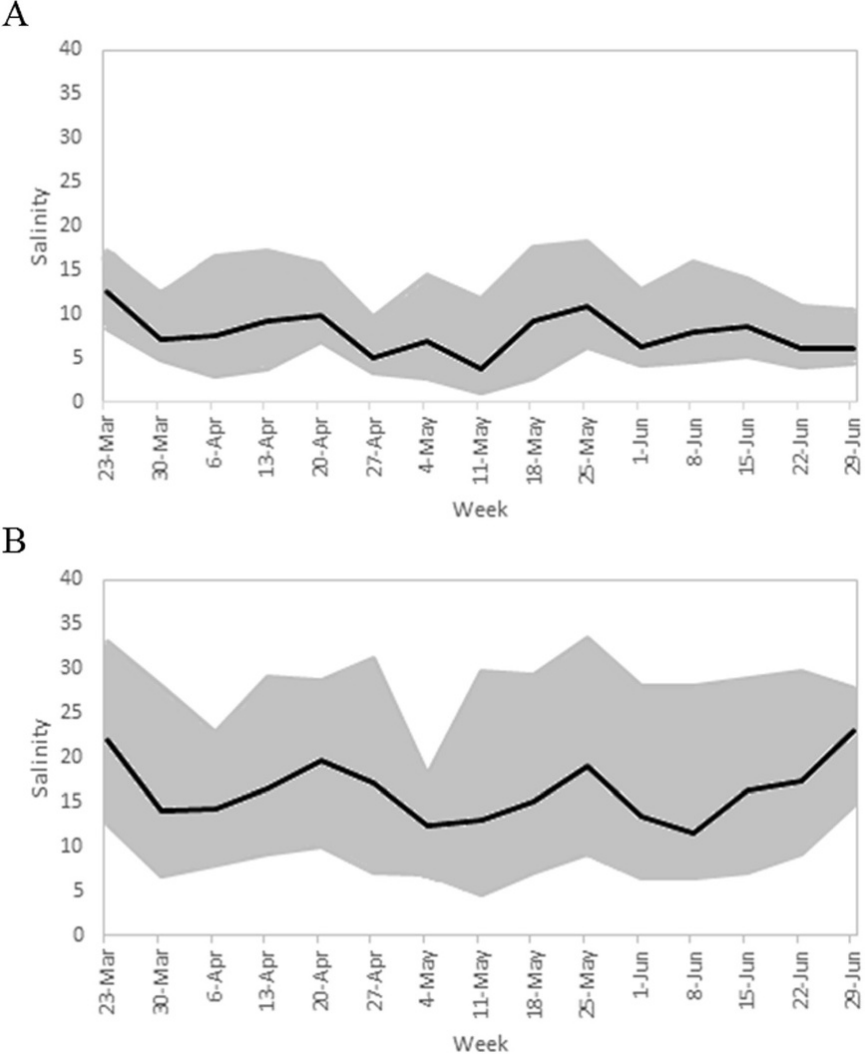
**Salinity at the ‘high’ and ‘low’ wild juvenile oyster collections sites. A)** Maximum, mean, and minimum salinity at low salinity site. **B)** Maximum, mean, and minimum salinity at high salinity site.

Shell substrate was deployed at a “high” salinity field site (27°10’58.2”N 80°12’22.2”W; mean salinity = 15.9, max = 33.5, min = 4.6) and at a “low” salinity field site (27°13’11.2”N 80°13’38.9”W; mean salinity = 8.0, max = 18.2, min = 1.0) in the St. Lucie River, Florida, on June 2, 2010. Water temperature, salinity, and percent dissolved oxygen were recorded every hour at both sites from March 23, 2010 until July 1, 2010 with a Sonde (YSI 600OMS V2). Over this time interval these two sites were significantly different in salinity (Figure 1, t = -38.6, df = 1397, p < 0.001). Mean water temperature was 26.5°C at the high salinity site and 27.4°C at the low salinity site. Mean dissolved oxygen was 83.1% at the high salinity site and 86.6% at the low salinity site. The temperature and dissolved oxygen did not differ significantly between the sites (p = 0.062, p = 0.091). Juvenile oysters (spat; 4 – 10 mm total length) were collected from the shell substrate on July 1, 2010. All soft tissue, including gill, mantle and adductor muscle, was archived for each individual in RNALater® (Ambion) after removing the visible digestive system. Within two weeks, the RNALater® was drained and the samples were archived at -80°C.

### RNA extraction

Approximately 30 mg tissue from each of four individuals per site was used for individual RNA extractions using Qiagen RNeasy Mini Kit (Qiagen, Valencia, CA) following the manufacturer's protocol. Total RNA from each sample was quantified using NanoDrop 8000 (ThermoScientific) and 5 μg from each of four individuals per site was pooled.

### 454 library prep and sequencing

The construction of two normalized cDNA libraries and 454 pyrosequencing was carried out at the W.M. Keck Center for Comparative and Functional Genomics, Roy J. Carver Biotechnology Center, University of Illinois at Urbana-Champaign. We chose to normalize the libraries in order to increase the likelihood that rare transcripts would be sequenced, leading to a more complete transcriptome with limited sequencing effort. For each library, messenger RNA was isolated from 10 μg of pooled total RNA with the Oligotex kit (Qiagen, Valencia, CA). The messenger RNA was then converted to a primary cDNA library with adaptors compatible with the 454 system using Multiplex Identifier (MID) tags to distinguish the two population pools [28]. The libraries were diluted to 1 × 10^6^ molecules/μL, pooled, and sequenced on a full plate using the 454 Genome Sequencer FLX + system according to the manufacturer’s instructions (454 Life Sciences, Branford, CT). Signal processing and base calling were performed using the bundled 454 Data Analysis Software v2.6.

### Transcriptome assembly and clustering

The two barcoded sets of reads were independently trimmed prior to assembly. Reads were trimmed from each end using a phred-scale quality score of 20 with fastq_quality_trimmer (FASTXToolkit). When the trimmer encountered a base pair with a quality less than 20, the closest read end was trimmed up to that base. Reads with less than 70% of the original length remaining were discarded. Trimmed reads were then imported into Newbler (gsAssembler, 454 Life Sciences, Roche Diagnostics). Any remaining adapters were trimmed, and reads were filtered against an *E. coli* database to remove contaminants. Reads were then assembled *de novo* using the default settings except for a minimum overlap length of 30 bp (default 40 bp). The quality of the initial assembly was evaluated by comparing assembly statistics to other published molluscan transcriptomes from 454 sequencing. Newbler reports consensus “contigs” using the overlap-layout-census (OLC) approach, which merges reads into contigs when their alignments overlap. Reads with no alignment overlap with other reads are denoted as singletons. Because singletons may belong to unique genes that were not highly expressed, they were included in further analysis. We defined a reference transcriptome as the combined set of 200 bp or longer contigs (consensus from multiple overlapping reads) and singletons and hereafter refer to these as “reftigs” (reference transcriptome sequences).

Large indels, highly polymorphic sequences, and other *de novo* assembly challenges can often lead to redundancy in sequences between singletons and contigs [29]. Particularly in highly polymorphic species such as oysters, alleles will often assemble into separate contigs or remain as singletons. This redundancy complicates downstream applications of the resulting transcriptome, such as gene expression analyses [30], because reads from some single copy genes will not map uniquely within the transcriptome and may be discarded. We assumed that the reftigs from the Newbler assembly included many oversplit loci, so to improve the transcriptome we consolidated redundant reftigs by clustering. Cd-hit [31] was used to cluster reftigs with various sequence identity thresholds ranging from permissive clustering at 80% to conservative clustering at 99% using a k-mer word size of 5 to 10 increasing incrementally with the threshold (e.g. word size 5 with threshold 80%).

Several approaches were used to evaluate whether clustering improved the transcriptome. For the 80% and 95% clustering results we compared statistics bearing on transcriptome quality including the percent of reftigs that were annotated, the distribution of annotation between contigs and singletons, and the number of osmoregulatory candidates (identified in *C. gigas*) recovered.

Additionally, we evaluated the two clustering results by comparing the proportion of Illumina reads from a barcoded individual that uniquely mapped to annotated reftigs based on a pilot RNA-seq experiment. The barcoded individual was from an oyster reef in Delaware Bay with a salinity regime ranging from 6.5 to 14.5. The mRNA from 30 mg of gill tissue was extracted using Dynabeads® mRNA DIRECT™ kit (Life Technologies). The library was prepared with NEBNext® mRNA Library Pep Reagent Set for Illumina® (New England BioLabs Inc.). The library constituted 16.25% of a single 100 bp Hi-Seq Illumina lane and was sequenced at the Biotechnology Resource Center Genomics Facility of Cornell University. The resulting reads were trimmed following the same procedure as the 454 reads, and any remaining adapters were clipped using fastx_clipper (FASTXToolkit). The remaining reads were then mapped to the annotated reftigs using BWA [32] with a mismatch edit distance of 0.005 and SAMtools [33] with only uniquely mapped reads retained.

### Annotation

To annotate the *de novo C. virginica* transcriptome assembly, reftigs were compared to NCBI's non-redundant (nr) protein sequence database that included the annotated proteins deduced from the *C. gigas* genome (May 2013), plus the Swiss-Prot and TrEMBL databases from the Uniprot protein knowledge base, using the BLASTx algorithm with an e-value cut-off of 10^-5^. Gene Ontology (GO; http://www.geneontology.org) annotation was retrieved from Uniprot. The annotated and unannotated reftigs were then compared with respect to the proportion of contigs and singletons as well as GC content in order to explore if unannotated reftigs may represent non-oyster contamination in the 454 sequences. The number of unique genes represented by the transcriptome was then identified by grouping reftigs that shared the same GenBank gene identifier.

We considered genes as osmoregulatory candidates if they were included in the 1,241 annotated genes found to be differentially expressed in *C. gigas* adults in response to six different salinity treatments when compared to a control salinity of 30 Table S21 in [17]. Additionally, we quantified the number of genes in the normalized libraries that were uniquely represented in one *C. virginica* population sample or the other by mapping the trimmed and filtered 454 read pools from ‘low’ and ‘high’ salinity samples back to the annotated reftigs from the 80%-clustered transcriptome using GSMapper (454 Life Sciences, Roche Diagnostics) with default settings. Enrichment of functional classes was tested at the level of genes, based on reftig annotation results described below, for two subsets compared to the entire annotated transcriptome: (1) all osmoregulatory candidates and (2) genes unique to each population. Enrichment tests used a Fisher’s exact test as implemented in TopGO from Bioconductor [34]. Genes that were unique to one of the two populations were identified as “asymmetric.” Results from enrichment tests were depicted in the context of the hierarchical structure of gene ontology terms in order to visualize the degree of functional integration among the most significantly enriched genes.

### Sequence comparisons with *C. gigas*

Simple sequence repeats and low complexity regions of the annotated reftigs were masked with RepeatMasker [35], using the rmblast search engine. Reftigs with masked regions were removed from analysis. The coding sequence reading frame for each remaining reftig was then predicted using ESTscan [36]. ESTscan was trained using the EMBL, RefSeq and UniGene clusters from the mollusk *Aplysia californica,* the most closely related species for which a full set of references were available at the time of this study. The matrices from this training were then used to predict coding sequences for the reftigs using a hidden Markov model [36]. The predicted coding sequence for each reftig was then used to analyze sequence divergence from *C. gigas*.

A local directory of *C. gigas* coding sequence for predicted proteins from the *C. gigas* genome was downloaded from http://gigadb.org/dataset/view/id/100030/sort/size and clustered with Cd-hit using the same parameters as for reftig clustering (sequence identity threshold = 0.8, word size = 5). Coding sequences that clustered were assumed to be paralogs and removed from analysis to reduce the bias that would occur with comparison of paralogs between *C. gigas* and *C. virginica*. The *C. virginica* reftigs were then compared against the *C. gigas* coding sequences using tBLASTx with intron linking disabled and an e-value cutoff of 10^-5^. Best hits were interpreted as putative ortholog pairs for analysis. Ortholog pairs were then run through a custom pipeline to align sequences using ClustalW [37] and calculate dN/dS ratios using the codeml function of paml [38]. The distribution of dN/dS values relative to ClustalW alignment length was evaluated before choosing to remove alignments less than 60% of the total reftig length.

### SNP discovery

The mapped 454 reads from both population samples were combined and aligned against the masked, annotated reftigs with mpileup of SAMtools [33]. SNPs were then identified using SNAPE-pooled [39] with a base quality average of 37 or greater, theta of 0.01, divergence of 0.1, flat prior and folded spectrum, and the SNP density for each contig was calculated.

## Results and discussion

### Assembly and clustering results

A total of 1,256,652 raw 454 reads included 718,009 from the high salinity population and 538,553 from the low salinity population. The raw reads are available through the National Center for Biotechnology Information Short Read Archive under accession numbers SRS502377 for the high salinity population and SRS502378 for the low salinity population. After trimming and filtering, 1,182,107 reads remained and were assembled into 28,939 contigs that contained 86.7% of the reads. The 128,083 unassembled reads were designated as singletons and included in further analysis. The combined contig and singleton set consisted of 157,022 reftigs. The assembly size for contigs alone was approximately 18,202,631 nucleotide bases, similar to other molluscan transcriptome assemblies based on 454 sequences (Table 1), and had an average contig length of 629.1 bases (N50 = 500 bases) and maximum contig length of 7,512 bases. The total transcriptome (contigs and singletons, 157,022 reftigs) was 51,918,466 nucleotides with an average length of 453.0 bases (N50 = 381 bases).

**Table 1:** **Assembly comparison to other molluscan transcriptomes sequenced using 454 technology**

Species	Normalized	Mean unfiltered read length (bp)	Unfiltered reads (n)	Assembler	% of filtered reads assembled	Contigs (n)	Mean contig length (bp)	Estimated total assembly (bp)*	Reference
*Mytilus edulis*	No	279	2,393,441	Celera, Cap3	92.0	74,622	645	48,131,190	[40]
*Bathymodiolus azoricus*	Yes	283	778,996	MIRA	74.8	75,407	509	38,382,163	[41]
*Hyriopsis cumingii*	No	296	981,302	Cap3	70.5	47,812	634	30,312,808	[42]
*Meretrix meretrix*	No	413	751,970	Cap3	87.3	35,205	679	23,904,195	[43]
*Patinopecten yessoensis*	Yes/No	313	970,422	Cap3	86.7	32,590	618	20,140,620	[44]
*Crassostrea virginica*	Yes	343	1,256,652	Newbler	86.8	28,939	629	18,202,631	Present study
*Ruditapes philippinarum*	Yes	–	457,717	MIRA3	–	32,606	546	17,802,876	[45]
*Chamelea gallina*	Yes	210	298,494	MIRA	–	39,750	352	13,992,000	[46]
*Laternula elliptica*	No	369	1,034,155	Newbler	33.9	18,290	535	9,785,150	[47]
*Crassostrea angulata*	No	309	555,215	Newbler	79.9	10,462	723	1,057,026	[48]
*Pinctada martensii*	No	349	434,650	Newbler	–	3,574	–	–	[49]
*Pinctada margaritifera*	No	234	276,738	TGICL	79.2	76,790	–	–	[50]

The assemblies are ordered in decreasing size of estimated total assembly. *This calculation is from the mean contig length and number of assembled contigs and is provided as a means to compare transcriptome size.

**Figure 2:**
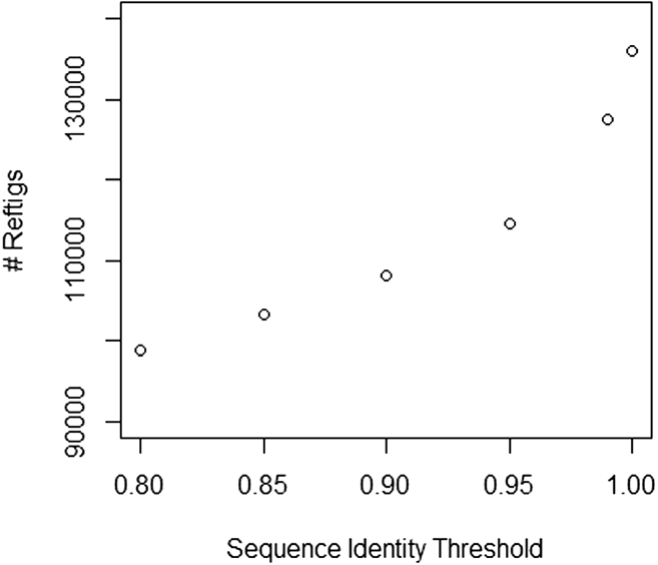
**Collapsing of reftigs with decreasing sequence identity threshold in Cd-hit.** As sequence identity threshold decreases in Cd-hit clustering, the number of reftigs decreases. The rate of reftig consolidation is highest from 1 to 0.95 but remains consistent from 0.95 to 0.90.

Given the high degree of polymorphism in oysters [17], clustering of assembled reftigs was explored as a method of consolidating alleles that remained apart after assembly. Consolidating alleles is an important consideration before using a transcriptome assembly as a reference for RNAseq expression analyses because oversplit alleles in the assembly will decrease the number of reads that map uniquely. A total of 136,000 reftigs (86.7%) were longer than 200 bp and used for clustering at different sequence identity thresholds. Comparing the change in total reftig number resulting from increasingly permissive clustering, the rate of reftig consolidation was initially rapid based on thresholds from 99% to 95%; then the rate of change slowed and was nearly constant between 95% and 80% (Figure 2). As the sequence identity threshold decreased, the ratio of contigs to singletons increased as expected if singletons were being clustered with greater frequency than contigs (Table 2).

**Table 2:** **Comparison of quality statistics for transcriptome assembly at unclustered, 95% and 80% sequence identity thresholds**

	Unclustered	95% sequence identity threshold	80% sequence identity threshold
Composition			
Total # Reftigs	136,000	114,716	98,729
% Contigs	16.4%	18.9%	20.5%
% Singletons	83.6%	81.1%	79.5%
Annotation			
Total reftigs annotated		58,811	50,736
% Reftigs annotated		51.3%	51.4%
% of Contigs		62.6%	64.3%
% of Singletons		48.6%	48.1%
# of Osmoregulation candidates		1014	1007
Sample illumina reads mapped to annotated transcriptome			
% Reftigs with mapped reads		86.7%	88.3%
% of Reads mapped uniquely		40.8%	42.8%

With more permissive clustering, the number of unique sequences in the transcriptome (reftigs) decreases and the proportion of non-singleton reftigs (contigs) increases. The clustering threshold has a trivial effect on the proportion of reftigs annotated and the number of osmoregulatory candidates recovered. With 42.8% of Illumina reads uniquely mapping, transcriptomes based on more permissive clustering have a greater percentage of annotated reftigs with reads mapped to them as well as a greater percentage of uniquely mapped reads.

Transcriptomes resulting from both the 95% and 80% sequence identity thresholds were annotated for comparison. Both transcriptomes had a similar percentage of reftigs successfully annotated, with a similar distribution of contigs and singletons. Likewise, 1,014 osmoregulatory candidates (see below) were obtained with the 95% threshold and this dropped by only seven candidate genes at the 80% threshold (Table 2). The large percentage (99.4%) of candidate genes that remain in the transcriptome at the 80% threshold compared to the 95% threshold suggests that any potential paralog clustering resulted in a minimal loss in the number of uniquely annotated genes, particularly osmoregulatory candidates.

We mapped 100 bp Illumina RNAseq reads from a single individual to the 95% and 80% transcriptomes to test whether oversplit alleles were consolidated by clustering. Relative to the 95% transcriptome, the 80% transcriptome had a higher percentage of annotated reftigs with mapped reads, but the effect was small (Table 2). In terms of the proportion of Illumina reads that mapped, two percent more reads mapped uniquely to the 80% transcriptome than the 95% transcriptome. The increase in the percent of uniquely mapped reads in the 80% transcriptome suggests that consolidation of allelic reftigs was achieved by clustering, resulting in more reads mapping uniquely. Based on these results the 80% threshold transcriptome was chosen for further analysis.

In one cluster examined in more detail, an original contig annotated as Heat Shock 70 kDA Protein 12 was ultimately clustered with three singletons. Two singletons clustered at the 95% threshold. The third singleton (330 bp) was unannotated at the 95% threshold. With a similarity of 84.55% estimated by Cd-hit, it was clustered with the contig and the other two singletons at the 80% threshold. An alignment between this third singleton and the original contig showed five indels ranging in size from 1 to 17 bp and two polymorphisms as the cause for the 84.55% sequence identity. We suspect these indels represent 454 sequencing error because they were partially shared across the three singletons, most of them would disrupt the reading frame, and they occurred within simple nucleotide repeats and low complexity sequence. Some of the SNPs present in the singletons may also be sequencing error but not obviously so; most were not adjacent to indels and they were already represented in the original contig. Therefore, clustering provided two distinct transcriptome improvements: (1) oversplit alleles were consolidated, facilitating downstream mapping to the transcriptome for RNAseq expression analysis, and (2) more of the 454 sequencing coverage was used to call SNPs.

The optimum balance between consolidating oversplit alleles and clustering paralogs or sequence errors is impossible to know because it depends on the distribution of allelic sequence differences relative to paralog differences in any particular species as well as the sequencing error rate. The comparative approach used here was ad hoc and took advantage of computational efficiencies when clustering consensus sequences from an assembly rather than exploring parameter values in separate assemblies. When no reference genome is available, this comparative empirical approach can be a valuable method of improving transcriptome quality.

### Annotation results

**Figure 3:**
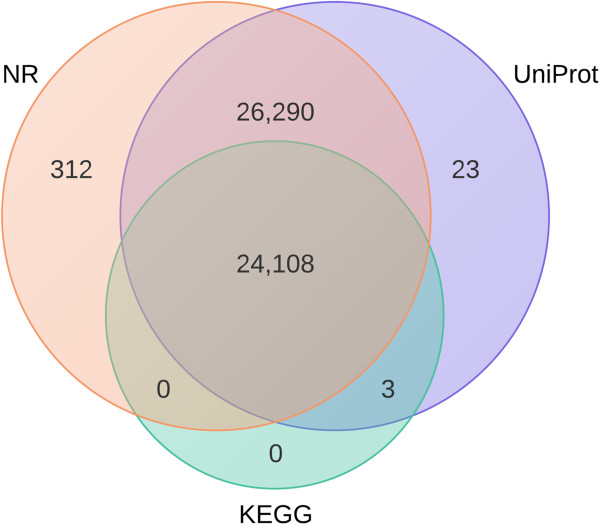
**Number of reftigs annotated by each database out of 50,736 total reftigs.** The Genbank nr database supplied 99.95% of annotations.

The BLASTx search against multiple databases provided annotation for 50,736 reftigs (51.4%) representing 20,249 unique GenBank accessions and 16,392 distinct putative proteins that we will refer to as genes. Only 0.05% of the annotations were achieved with a database other than GenBank nr (Figure 3). Reftigs that did not have a BLASTx match with an e-value smaller than 10^-5^ from any database was designated as unannotated.

Of 16,392 distinct genes, 8,161 are represented by a single reftig. The number of reftigs per gene ranges from 1 to 470 with only 13 genes represented by 100 or more reftigs. Gene duplication and large gene families, particularly in the *C. gigas* genome from which 89% of our annotations were identified, are the primary reasons for the large number of reftigs per “gene”. For example, the 456 reftigs identified as the gene “tripartite motif-containing protein 2” from *C. gigas* were annotated from 201 unique GenBank accessions. For *C. gigas*, these different GenBank accessions represent different coding sequence locations within the genome. Therefore, we define a “gene” here as a protein product, which often represents large gene families.

Gene Ontology (GO) terms were assigned to 36,924 of the annotated reftigs, representing 11,583 putative genes, based on sequence similarity to known proteins in the UniProt databases. Annotated reftig sequences have been archived and are accessible through FigShare (http://dx.doi.org/10.6084/m9.figshare.873865)[51].

**Figure 4:**
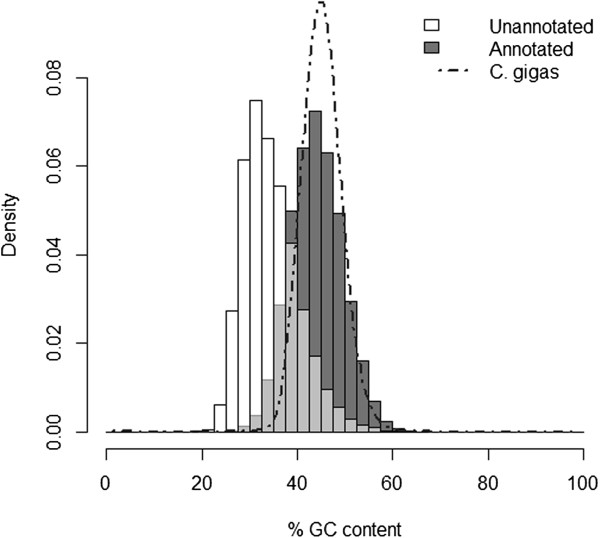
**GC content of annotated and unannotated portions of transcriptome and** ***C. gigas.*** GC content of the annotated portion of the transcriptome is higher than in the unannotated portion, suggesting potential contamination from other species or the presence of other RNA types in the sequencing. The GC content of the annotated portion mirrors the GC content found in the coding sequences of *C. gigas*.

A consequence of whole animal extractions and normalizing the libraries was the increased potential to sequence non-oyster transcripts, such as bacteria and algae. Singletons made up ~74% of the reftigs in the full (annotated and unannotated) 80% transcriptome and less than half of the singletons were successfully annotated. Other studies using 454 sequencing also have described singletons as comprising a large proportion of their transcriptome (e.g. 81.7% [52]; 58.5% [29]; 55.3% [53]) with a subset getting annotated. Singletons are the inevitable consequence of assembling transcripts with low coverage, so they are not necessarily indicative of contamination. However, here the mean GC content for annotated reftigs was 44% (SD = 5.4%), very similar to the 45.2% (SD = 4.3%) average for *C. gigas* coding sequences (Figure 4). In contrast, the unannotated portion of the *C. virginica* transcriptome had a significantly different mean GC content of 34.5% (SD = 5.8%) (Figure 4; t = 266, df = 973455, p < 0.001). The difference in GC content provides very strong evidence that many of the unannotated reftigs (both contigs and singletons) came from other organisms such as prokaryotes or protozoa. Without the benefit of the *C. gigas* reference genome for annotation and GC content comparison, *de novo* analysis of the eastern oyster transcriptome generated here would be highly compromised by contaminants.

### Osmoregulation candidate genes

Of the 1,241 osmoregulatory candidate genes identified in *C. gigas* [17], 1,007 (81.2%) were identified in the *C. virginica* transcriptome based on 9,307 reftigs (18.3% of all annotated reftigs) (Additional file 1: Table S1). The *C. gigas* candidates were identified experimentally based on differentially expressed genes between different salinity treatments of adult oysters [17], while we obtained these transcripts in wild juveniles. Thus, life stage is one factor that could help account for the *C. gigas* candidates that were not obtained in our samples. Additionally, *C. virginica* and *C. gigas* have mostly overlapping but slightly different salinity tolerances with *C. virginica* having lower mortality rates than *C. gigas* at low salinity and vice versa at higher salinities [54].

Fifty-nine osmoregulatory candidate genes were identified from only the high salinity population and 56 were identified from only low salinity, together representing 11.4% of the candidate genes. These asymmetrically expressed candidate genes were mostly cases with 1x coverage (1:0 asymmetry), but 3 genes (2.6%) had an asymmetry ratio of 5:0 or greater.

Ignoring candidate status, 4,053 of the 16,392 annotated *C. virginica* genes (24.7%) were identified from only one of the two populations. Of these, 2,185 were found only in the high-salinity population, including 1,431 genes (8.7%) with 1:0 asymmetry and 74 genes (0.5%) with 5:0 or greater asymmetry. An additional 1,868 genes were found only in the low-salinity population, including 1,355 (8.3%) with a 1:0 asymmetry and 31 genes (0.2%) with 5:0 or greater asymmetry. A total of 105 genes (2.6%) from the two populations had 5:0 or greater asymmetry.

The fact that 24.7% of all genes showed asymmetry, while only 11% of osmoregulatory candidates did so, suggests that there may be many biological processes leading to population-specific expression in addition to the stochasticity expected with low-expression genes. Also, given that buffering against osmotic stress is a chronic physiological need for oysters, lower asymmetry among osmoregulatory candidates might reflect the proportion of genes within this functional category that have constitutive expression across salinities.

**Figure 5:**
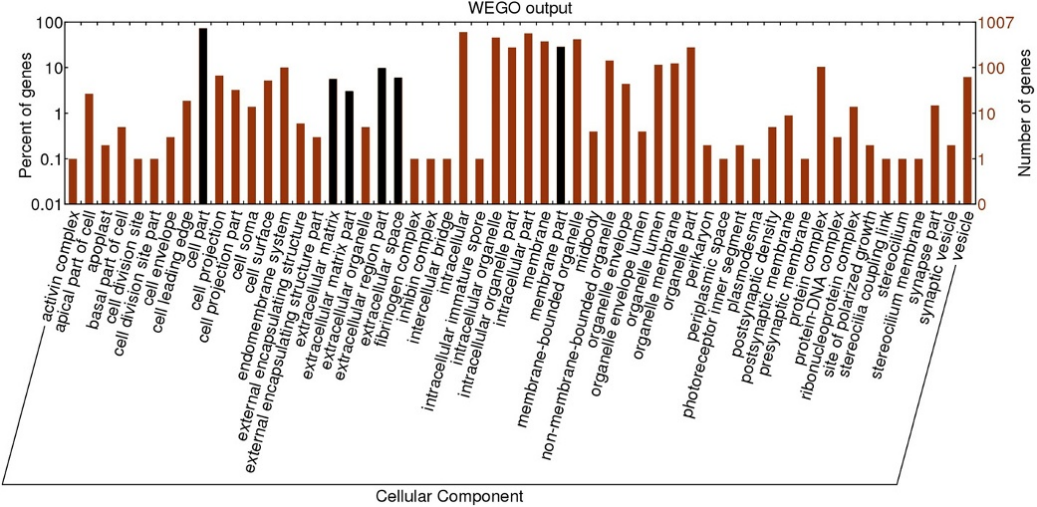
**Distribution of level 3 Cellular Component GO terms for the osmoregulatory candidate genes.** Black bars indicate the terms or the parents of GO terms that are significantly enriched in the osmoregulatory candidate genes compared to the complete set of annotated genes.

**Figure 6:**
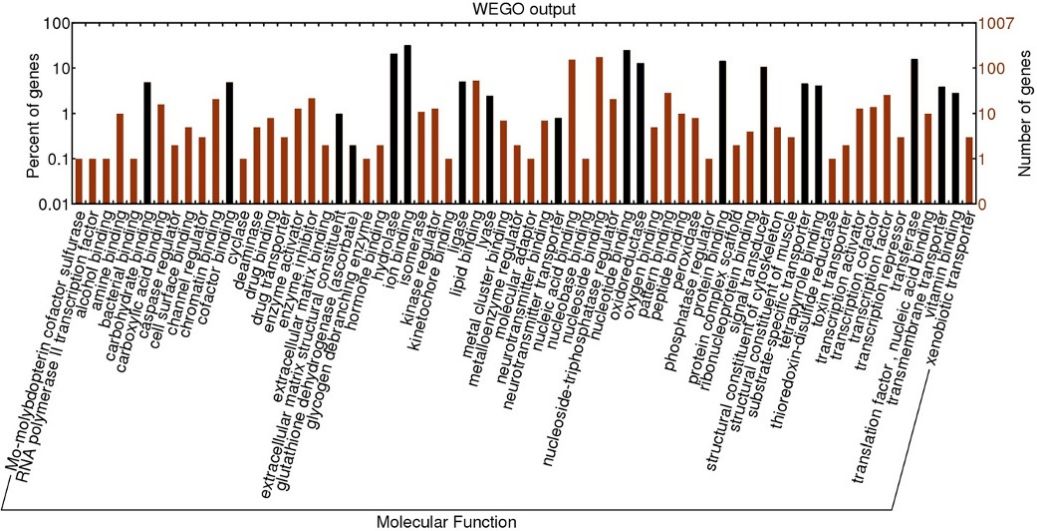
**Distribution of level 3 Molecular Function GO terms for the osmoregulatory candidate genes.** Black bars indicate the terms or the parents of GO terms that are significantly enriched in the osmoregulatory candidate genes compared to the complete set of annotated genes.

It is difficult to know how much asymmetry to expect by chance for genes with a given level of expression in normalized libraries. However, enrichment of functional categories within the set of population-specific genes is not expected from stochastic variation in library normalization or read coverage. Our prediction was that among asymmetric genes, annotations related to osmoregulatory function should be the most highly enriched relative to the frequency of functional ontologies in the overall annotated transcriptome. We initially built a frame of reference by testing for functional enrichment among the entire set of osmoregulatory candidate genes in *C. virginica* and found 12 cellular component GO terms and 86 molecular function GO terms significantly enriched compared to the complete annotated gene set (Figures 5 and 6, Additional file 2: Tables S2 and S3). For cellular components, the ‘extracellular region’ , ‘plasma membrane’ and ‘membrane’ components were among the significantly enriched terms (Figure 5 and Additional file 2: Table S2). At the level of molecular function, ‘catalytic’ activities, ‘binding’ functions, ‘electron carrier’ activities, ‘transporter’ activities and ‘molecular transducer’ activities were among the significantly enriched terms (Figure 6, Additional file 2: Table S3). These enriched GO terms serve to functionally characterize the osmoregulatory candidates on the whole and therefore might be indicators of osmoregulatory function in additional enrichment tests when found in concert.

As predicted, the overall group of asymmetric genes (24.7% of all genes) showed significantly enriched ontologies relating to osmoregulatory function, as indicated by similarities with enriched GO terms in the total set of osmoregulatory candidate genes. Interestingly, the enriched GO terms were only partially overlapping in the low versus high salinity population, and relative enrichment magnitudes shifted among GO terms. In the low salinity population at the level of cellular components, the strongest result among 12 significantly enriched ontologies included ‘integral to membrane’ (GO:0016021, p = 0.00037) and ‘intrinsic to membrane’ (GO:0031224, p = 0.00058), two ontologies nested within ‘membrane’ components, the level-three GO term enriched among osmoregulatory candidates (Additional file 2: Table S4). Transmembrane channels are important in maintaining cell volume in response to hypoosmotic stress. For example, Ca^2+^ channels are upregulated in hypoosmotic stress and osmolytes such as taurine are taken up through high affinity transport systems that may involve transmembrane proteins [18]. Additional terms such as ‘cell periphery’ (GO:0071944, p = 0.0033), ‘plasma membrane part’ (GO:0044459, p = 0.00624) and ‘plasma membrane’ (GO:0005886, p = 0.00815) were terms significantly enriched both in the asymmetric low salinity genes and the full candidate gene set (Additional file 2: Tables S2 and S4). In general, however, the cellular component terms most strongly enriched in the full set of osmoregulatory candidates, extracellular region and its ‘children’ terms, were not enriched in genes expressed solely at low salinity in *C. virginica*.

In contrast to the low population, the most significant functional enrichment at the level of cellular components in the high population was ‘extracellular region’ (GO:0005576, p = 6.4e-08). This term refers to the gene products that are secreted from the cell but retained in the interstitial fluid or hemolymph, and it was also the most significantly enriched for the full osmoregulatory candidate gene set (Additional file 2: Table S2, Figure 5). While this parent GO term had the highest level of enrichment among the *C. gigas* genes experimentally associated with salinity treatments [19] (Additional file 2: Table S2), it is also likely to include immune response genes responding to the larger disease burden found in oysters from high salinity [19, 55]. Several additional GO terms were significantly enriched both in the asymmetric high salinity genes and in the full candidate gene set including ‘intrinsic to membrane’ (GO:0031224, p = 6.4e-06) and ‘plasma membrane’ (GO:0005886, p = 1.3e-05) (Additional file 2: Tables S2 and S5).

At the molecular functions GO level, both the high and low salinity populations showed the strongest significant enrichments related to DNA replication and transcription/translation (Additional file 2: Tables S6 and S7). For the low salinity population, many of the other significantly enriched molecular function ontologies (Additional file 2: Table S6) were ‘children’ terms of those significantly enriched both for osmoregulatory genes and for unique low salinity genes (Additional file 2: Tables S3 and S6, Figure 6). For example, ‘G-protein coupled receptor activity’ (GO:0004930, p = 6.6e-07) is a ‘child’ term of ‘receptor activity’ and ‘aspartic-type peptidase activity’ (GO:0004190, p = 2.2e-06) is a ‘child’ term of ‘hydrolase activity.’ These enriched functions match predictions that the phosphorylation of plasma membrane proteins and the hydrolysis of peptides are part of the physiological response to osmotic stress. For the high salinity population, significant enrichment was found for potential osmoregulatory terms related to ‘substrate-specific transporter’ and ‘transmembrane transporter’ activities such as ‘receptor activity’ (GO:0004872, p = 7.8e-06), ‘gated channel activity’ (GO:0022839, p = 0.00022), and ‘ion gated channel activity’ (GO:0022839, p = 0.0022) (Additional file 2: Table S7). These enrichment results are consistent with expectations for differential expression of osmoregulatory genes by juvenile eastern oysters from different salinity regimes. Furthermore, it confirms the functional relevance in *C. virginica* of osmoregulatory candidates identified in *C. gigas*.

At the level of reftigs, rather than genes, among those with annotations linked to osmoregulatory function in *C. gigas* (9703 reftigs), 57.3% showed expression in only one of the two populations. This high frequency of asymmetry is in striking contrast to the 11% asymmetry measured at the gene level among osmoregulatory candidates. One possible explanation for this pattern is that asymmetric reftigs represent differentially expressed splice variants of genes expressed by both populations. This hypothesis will be testable with the benefit of this transcriptome as a reference for RNA-seq analyses.

### SNP Discovery and dN/dS ratio with C. gigas

**Figure 7:**
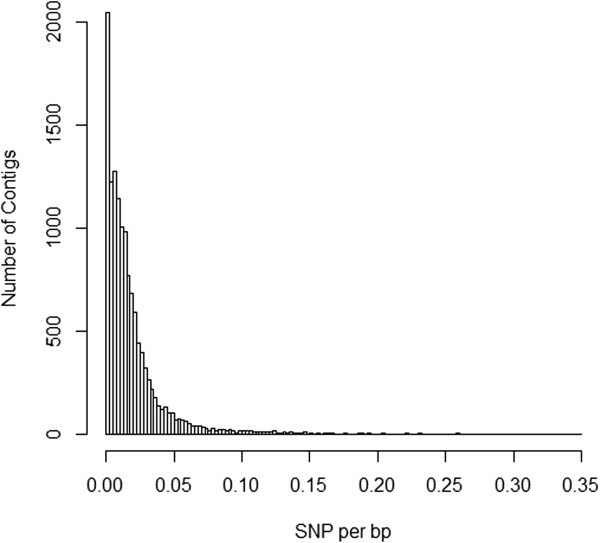
**The distribution of SNP density per base pair within annotated contigs from the 80% clustered transcriptome.**

The transcriptome we present here provides the most comprehensive estimate of polymorphism to date for *C. virginica*. Among 13,108 annotated contigs, there was 12,355,575 bp of aligned sequence within which 218,777 SNPs were identified. Average SNP density was 0.0185 per base pair with a standard deviation of 0.0238 (Figure 7). Ninety percent of contigs had at least one predicted SNP. This SNP density falls within the range previously reported for the eastern oyster. Quilang et al. [56] found a rate of 0.0059 SNPs/bp from 4,688 EST sequences. In contrast, Zhang and Guo [57] estimated 0.042 SNPs/bp based on resequencing 6.8 kb of ESTs. Similarly, a single gene study of serine protease inhibitor reported an overall SNP frequency of 0.044/bp [58]. For comparison, SNP density averaged across all exons in wild-caught *C. gigas* was 0.0102 per bp [17]. Our finding is therefore consistent with previous estimates of nucleotide heterozygosity in *C. virginica* and tentatively supports the contention that this species is more polymorphic than *C. gigas* [57].

**Figure 8:**
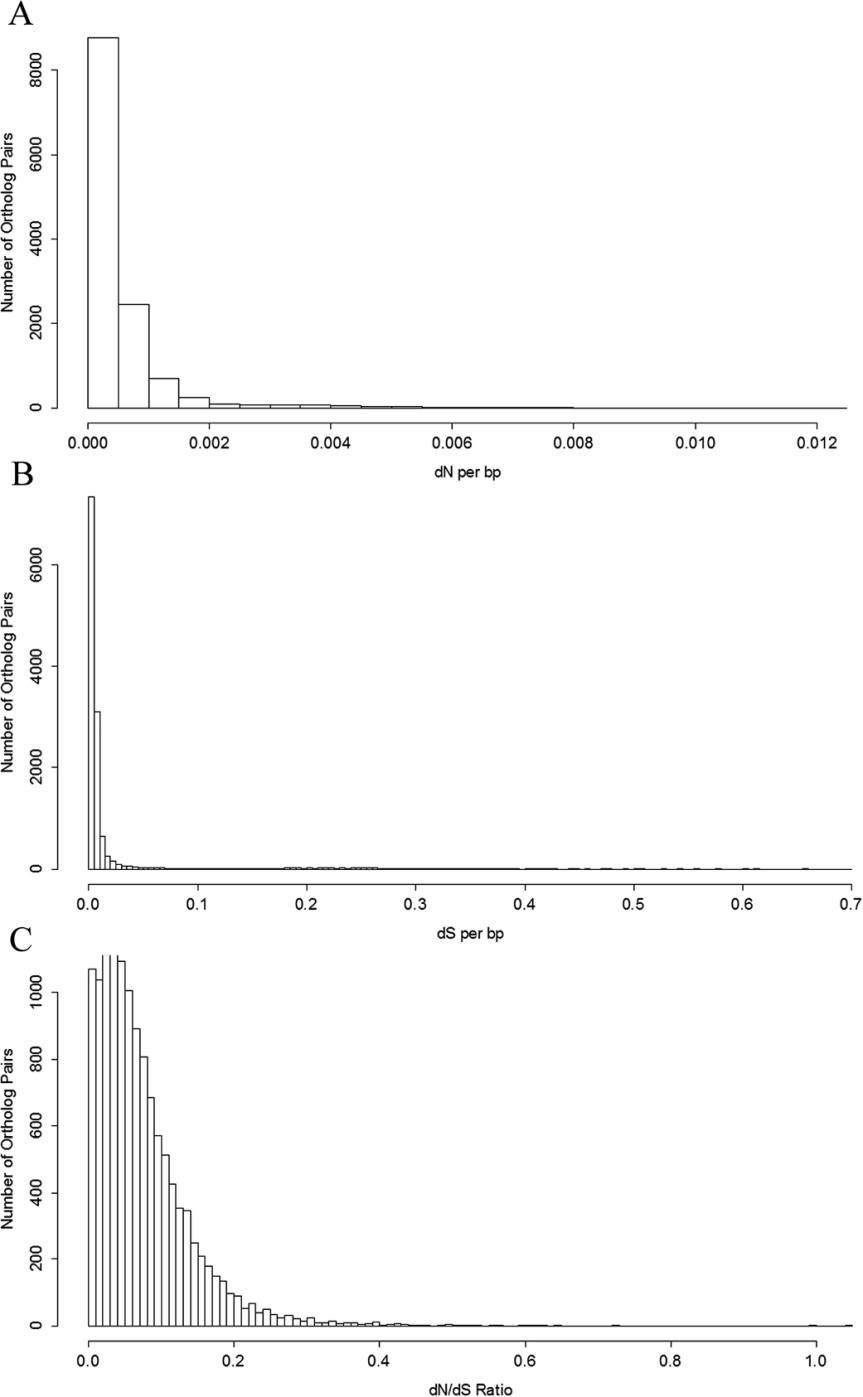
**Distribution of (A) dN, (B) dS and (C) dN/dS ratio values.** dN and dS show similar distribution shapes with the number of synonymous substitutions much larger than the number of nonsynonymous substitutions. Most ortholog pairs had a dN/dS ratio below 0.2 indicating a strong role for purifying selection on oyster peptide sequences.

Quantifying genomic patterns of divergence between *C. virginica* and *C. gigas* can help assess the relevance of discoveries in one species with respect to the other. Also, the ratio of substitution rates at nonsynonymous and synonymous sites can help to identify genes undergoing positive selection. After various filtering steps to remove potential artifacts and paralog gene pairs, 26,102 annotated reftigs from *C. virginica* were paired with an ortholog from *C. gigas*. Estimates for the number of nonsynonymous substitutions per nonsynonymous site ranged from near 0 to 0.012/bp per ortholog gene pair (Figure 8A) while the number of synonymous substitutions per synonymous site ranged from 0.0003 to 0.66/bp (Figure 8B). The mean dN/dS ratio of 0.074 (SD = 0.066, Figure 8C) indicates a pervasive role for purifying selection maintaining similar amino acid sequences. The mean protein similarity was 76.8%, and the mean nucleotide similarity was 74.2%. It is possible that these divergence estimates between the oyster congeners are biased downward because filtering steps (see Methods) inevitably removed some more divergent ortholog pairs.

This degree of purifying selection provides some confidence that functional candidate genes identified in *C. gigas* will often be applicable to *C. virginica*, at least as a starting point. At the same time, transcriptomes in these two species are probably too diverged to expect *C. gigas* genomic reference sequences to help with *C. virginica* bioinformatics. A simulation study by Vijay et al. [59] demonstrated that reference genomes with average nucleotide sequence divergence up to 15% can help improve transcriptome assemblies while with greater divergence there was no improvement over a *de novo* assembly. Similarly, the potential for a heterologous reference genome to provide improved RNA-seq analyses, relative to a *de novo* transcriptome assembly, was determined to be at nucleotide sequence divergences less than 15% [59].

## Conclusions

The goal of our study was to assemble and annotate the *C. virginica* transcriptome with particular focus on potential osmoregulatory genes. Largely with the benefit of the Pacific oyster genome, we assigned provisional annotations to 50,736 reftigs representing over 16,000 putative proteins. More than 80% of the osmoregulatory gene candidates identified in *C. gigas* experiments with adults were identified here in wild juvenile samples from different salinities. The low dN/dS between *C. virginica* and *C. gigas* indicates purifying selection in the coding regions of orthologous genes and provides justification that genes identified as osmoregulatory in *C. gigas* are likely to maintain the same function in *C. virginica.* Even stronger justification is reported for a subset of osmoregulatory candidates that were expressed in only one of the two different salinity populations. Genes with an asymmetric expression pattern across the salinity gradient were significantly enriched for functions that may be related to osmoregulation, consistent with these genes having osmoregulatory functions in *C. virginica*.

Additionally, we have demonstrated that permissive clustering of contig and singleton sequences may improve downstream applications of assembled transcriptomes. In some *de novo* transcriptome assembly studies, the singleton reftigs are discarded and only the contigs are analyzed. Such a stringent filter, if applied here to *C. virginica*, would have eliminated 37,717 singletons that were successfully annotated. The goal of clustering is to keep the singletons and reduce redundancy across reftigs that can result from *de novo* assembly challenges due to factors such as sequencing error and high levels of polymorphism. Several studies employing programs such as Cd-hit to cluster sequences based on similarity used a threshold of 95% similarity [7, 49]. We explored a range of threshold values from 99% down to the lower limit of the algorithm at 80%. The improvement in uniquely mapped reads may be beneficial for downstream applications, depending on experimental goals. For RNA-seq experiments, a greater number of uniquely mapped reads means that a greater percentage of data can be retained for the estimation of expression. Future development of these clustering procedures should focus on evaluating trade-offs, particularly with respect to the incorporation of sequencing error at more permissive clustering thresholds.

Finally, we have provided a valuable set of resources for eastern oyster research. We have annotated 50,736 reftigs, doubling the 48,183 *C. virginica* transcriptome contig sequences provided by Zhang et al. [7]. After careful filtering of these reftigs, we identified 218,777 candidate SNPs for use in genetic mapping or for population analyses. The 1,007 candidate genes for osmoregulation identified here will provide a reference for future studies on the molecular basis of osmoregulation in *C. virginica*, phenotypically plastic responses to salinity stress, and patterns of selective differentiation across heterogeneous environments.

### Availability of supporting data

The data sets supporting the results of this article are available in the National Center for Biotechnology Information Short Read Archive, accession numbers SRS502377 (http://www.ncbi.nlm.nih.gov/biosample/SRS502377) and SRS502378 (http://www.ncbi.nlm.nih.gov/biosample/SRS502378), and in FigShare (http://dx.doi.org/10.6084/m9.figshare.873865).

## Electronic supplementary material

Additional file 1: Table S1: Annotation of osmoregulatory candidate transcriptome reftigs from C. virginica. Each row is an osmoregulatory candidate reftig identified from the C. virginica transcriptome. The identification as an osmoregulatory candidate is from the match between the GenBank nr description (column 3) of the annotated reftig to the description of an osmoregulatory candidate identified by Zhang et al. [17] in the C. gigas genome. Information provided in the table for each reftig are the reftig length, GenBank nr description, nr e-value, UniProt match, UniProt e-value, UniProt ID, KEGG ID, and nucleotide sequence. (XLSX 2 MB)

Additional file 2: Table S2: Significantly enriched gene ontologies for cellular components in osmoregulatory candidate genes. Osmoregulatory candidate genes were compared to the complete set of annotated genes, ordered by functional category. The p-value is derived from a Fisher’s exact test implemented in topGO from Bioconductor. Indentations represent the ‘parent’:‘child’ tiered relationship of GO terms with deeper indentations representing more specific terminology relative to the boldface level-three ‘parent’ terms shown as enriched in Figure 5. **Table S3.** Significantly enriched gene ontologies for molecular functions in osmoregulatory candidate genes. Osmoregulatory candidate genes were compared to the complete set of annotated genes, ordered by functional category. The p-value is derived from a Fisher’s exact test implemented in topGO from Bioconductor. Indentations represent the ‘parent’:‘child’ tiered relationship of GO terms with deeper indentations representing more specific terminology relative to the boldface level-three ‘parent’ terms shown as enriched in Figure 6. **Table S4.** Significantly enriched gene ontologies for cellular components from the low salinity population. Significantly enriched gene ontologies in 1:0 asymmetric genes from the low salinity population are ordered by p-value. The p-value is derived from a Fisher’s exact test implemented in topGO from Bioconductor. **Table S5.** Significantly enriched gene ontologies for cellular components from the high salinity population. Significantly enriched gene ontologies in 1:0 asymmetric genes from the high salinity population are ordered by p-value. The p-value is derived from a Fisher’s exact test implemented in topGO from Bioconductor. **Table S6.** Significantly enriched gene ontologies for molecular function from the low salinity population. Significantly enriched gene ontologies in 1:0 asymmetric genes from the low salinity population are ordered by p-value. The p-value is derived from a Fisher’s exact test implemented in topGO from Bioconductor. **Table S7.** Significantly enriched gene ontologies for molecular function from the high salinity population. Significantly enriched gene ontologies in 1:0 asymmetric genes from the high salinity population are ordered by p-value. The p-value is derived from a Fisher’s exact test implemented in topGO from Bioconductor. (DOCX 52 KB)
