# Supplementary material for: Transcriptomic analysis of candidate osmoregulatory genes in the eastern oyster Crassostrea virginica
Source: BMC Genomics. 2014 Jun 20;15(1):503. doi: 10.1186/1471-2164-15-503 (PMC4101419; doi:10.1186/1471-2164-15-503)
Supplement: Supplementary file 2 — Additional file 2: Table S2: Significantly enriched gene ontologies for cellular components in osmoregulatory candidate genes. Osmoregulatory candidate genes were compared to the complete set of annotated genes, ordered by functional category. The p-value is derived from a Fisher’s exact test implemented in topGO from Bioconductor. Indentations represent the ‘parent’:‘child’ tiered relationship of GO terms with deeper indentations representing more specific terminology relative to the boldface level-three ‘parent’ terms shown as enriched in Figure 5. Table S3. Significantly enriched gene ontologies for molecular functions in osmoregulatory candidate genes. Osmoregulatory candidate genes were compared to the complete set of annotated genes, ordered by functional category. The p-value is derived from a Fisher’s exact test implemented in topGO from Bioconductor. Indentations represent the ‘parent’:‘child’ tiered relationship of GO terms with deeper indentations representing more specific terminology relative to the boldface level-three ‘parent’ terms shown as enriched in Figure 6. Table S4. Significantly enriched gene ontologies for cellular components from the low salinity population. Significantly enriched gene ontologies in 1:0 asymmetric genes from the low salinity population are ordered by p-value. The p-value is derived from a Fisher’s exact test implemented in topGO from Bioconductor. Table S5. Significantly enriched gene ontologies for cellular components from the high salinity population. Significantly enriched gene ontologies in 1:0 asymmetric genes from the high salinity population are ordered by p-value. The p-value is derived from a Fisher’s exact test implemented in topGO from Bioconductor. Table S6. Significantly enriched gene ontologies for molecular function from the low salinity population. Significantly enriched gene ontologies in 1:0 asymmetric genes from the low salinity population are ordered by p-value. The p-value is derived from a Fisher’s exac [file 12864_2013_6237_MOESM2_ESM.docx]

**Supplementary Table 2. Significantly enriched gene ontologies for cellular components in osmoregulatory candidate genes.**

Osmoregulatory candidate genes were compared to the complete set of annotated genes, ordered by functional category. The p-value is derived from a Fisher’s exact test implemented in topGO from Bioconductor. Indentations represent the ‘parent’:‘child’ tiered relationship of GO terms with deeper indentations representing more specific terminology relative to the boldface level-three ‘parent’ terms shown as enriched in Fig. 5.

| GO ID | Term | p-value |
| --- | --- | --- |
| GO:0005576 | extracellular region | 4.6e-16 |
| GO:0044421 | **extracellular region part** | 1.9e-15 |
| GO:0031012 | **extracellular matrix** | 7.9e-14 |
| GO:0044420 | **extracellular matrix part** | 1.7e-14 |
| GO:0005578 | proteinaceous extracellular matrix | 1.1e-12 |
| GO:0005581 | collagen | 1.2e-11 |
| GO:0005604 | basement membrane | 0.0033 |
| GO:0005615 | **extracellular space** | 7.4e-07 |
| GO:0005623 | cell | --------- |
| GO:0044464 | **cell part** | --------- |
| GO:0071944 | cell periphery | 0.0011 |
| GO:0005886 | plasma membrane | 0.0017 |
| GO:0044459 | plasma membrane part | 0.0032 |
| GO:0016020 | membrane | --------- |
| GO:0044424 | **membrane part** | --------- |
| GO:0031224 | intrinsic to membrane | --------- |
| GO:0031225 | anchored to membrane | 0.0076 |

**Supplementary Table 3. Significantly enriched gene ontologies for molecular functions in osmoregulatory candidate** **genes.**

Osmoregulatory candidate genes were compared to the complete set of annotated genes, ordered by functional category. The p-value is derived from a Fisher’s exact test implemented in topGO from Bioconductor. Indentations represent the ‘parent’:‘child’ tiered relationship of GO terms with deeper indentations representing more specific terminology relative to the boldface level-three ‘parent’ terms shown as enriched in Fig. 6.

| GO.ID | Term | p-value |
| --- | --- | --- |
| GO:0003824 | catalytic activity | 6.3e-12 |
| GO:0016491 | **oxidoreductase activity** | 1.2e-16 |
| GO:0004497 | monooxygenase activity | 1.6e-14 |
| GO:0016705 | oxidoreductase activity acting on paired donors with incorporation or reduction of  molecular oxygen | 5.3e-14 |
| GO:0016713 | oxidoreductase activity acting on paired donors, with incorporation or reduction ofmolecular oxygen, reduced iron-sulfur protein as one donor, and incorporation of oneatom of oxygen | 0.00068 |
| GO:0018685 | alkane 1-monooxygenase activity | 0.00068 |
| GO:0031545 | peptidyl-proline 4-dioxygenase activity | 0.00028 |
| GO:0031543 | peptidyl-proline dioxygenase activity | 0.00077 |
| GO:0008392 | arachidonic acid epoxygenase activity | 0.00771 |
| GO:0016712 | oxidoreductase activity acting on paired donors, with incorporation or reduction ofmolecular oxygen, reduced flavin or flavoprotein as one donor, and incorporation of oneatom of oxygen | 3.5e-07 |
| GO:0070330 | aromatase activity | 1.1e-06 |
| GO:0016715 | oxidoreductase activity acting on paired donors, with incorporation or reduction ofmolecular oxygen, reduced ascorbate as one donor, and incorporation of one atom ofoxygen | 0.00014 |
| GO:0004500 | dopamine beta-monooxygenase activity | 0.00028 |
| GO:0016627 | oxidoreductase activity acting on CH-CH group of donors | --------- |
| GO:0017150 | tRNA dihydrouridine synthase activity | 0.00253 |
| GO:0016614 | oxidoreductase activity acting on CH-OH group of donors | 0.00335 |
| GO:0016618 | hydroxypyruvate reductase activity | 0.00771 |
| GO:0030267 | glyoxylate reductase (NADP) activity | 0.00771 |
| GO:0030613 | oxidoreductase activity acting on phosphorus or arsenic in donors | 0.00771 |
| GO:0030614 | oxidoreductase activity acting on phosphorus or arsenic as donors, disulfide as acceptor | 0.00771 |
| GO:0050610 | methylarsonate reductase activity | 0.00771 |
| GO:0016641 | oxidoreductase activity acting on CH-NH2 group of donors, oxygen as acceptor | --------- |
| GO:0052597 | diamine oxidase activity | 0.00771 |
| GO:0052598 | histamine oxidase activity | 0.00771 |
| GO:0052599 | methylputrescine oxidase activity | 0.00771 |
| GO:0052600 | propane-1,3-diamine oxidase activity | 0.18 |
| GO:0016682 | oxidoreductase activity acting on diphenols and related substances as donors, oxygen as  acceptor | 0.00807 |
| GO:0051213 | dioxygenase activity | 0.00919 |
| GO:0016787 | **hydrolase activity** | --------- |
| GO:0004725 | protein tyrosine phosphatase activity | 3.5e-06 |
| GO:0016791 | phosphatase activity | 1.8e-05 |
| GO:0042578 | phosphoric ester hydrolase activity | 0.00023 |
| GO:0004721 | phosphoprotein phosphatase activity | 0.00107 |
| GO:0008833 | deoxyribonuclease IV (phage-T4-induced) activity | 0.00771 |
| GO:0016740 | **transferase activity** | --------- |
| GO:0016763 | transferase activity transferring pentosyl groups | 1.5e-05 |
| GO:0003950 | NAD+ ADP-ribosyltransferase activity | 5.5e-05 |
| GO:0016757 | transferase activity transferring glycosyl groups | 0.00232 |
| GO:0047273 | galactosylgalactosylglucosylceramide beta-D-acetylgalactosaminyltransferase  activity | 0.00771 |
| GO:0016769 | transferase activity transferring nitrogenous groups | 0.00451 |
| GO:0008483 | transaminase activity | 0.00451 |
| GO:0016874 | **ligase activity** | --------- |
| GO:0004812 | aminoacyl-tRNA ligase activity | 0.00085 |
| GO:0016875 | ligase activity forming carbon-oxygen bonds | 0.00085 |
| GO:0016876 | ligase activity forming aminoacyl-tRNA and related compounds | 0.00085 |
| GO:0016829 | **lyase activity** | --------- |
| GO:0004794 | L-threonine ammonia-lyase activity | 0.00771 |
| GO:0005488 | binding | --------- |
| GO:0046906 | **tetrapyrrole bonding** | 3.3e-12 |
| GO:0020037 | heme binding | 1.7e-12 |
| GO:0043167 | **ion binding** | 0.00012 |
| GO:0043169 | cation binding | --------- |
| GO:0005506 | iron ion binding | 1.1e-10 |
| GO:0005507 | copper ion binding | 4.3e-07 |
| GO:0043168 | anion binding | 8.7e-05 |
| GO:0030170 | pyridoxal phosphate binding | 0.00023 |
| GO:0070403 | NAD+ binding | 0.00113 |
| GO:0031406 | carboxylic acid binding | 0.00327 |
| GO:0031418 | L-ascorbic acid binding | 0.00451 |
| GO:0048037 | **cofactor binding** | 3.4e-05 |
| GO:0030246 | **carbohydrate binding** | 0.00020 |
| GO:0048029 | monosaccharide binding | 0.00802 |
| GO:0036094 | **small molecule binding** | 0.00120 |
| GO:0019842 | vitamin binding | 0.00116 |
| GO:0000166 | nucleotide binding | 0.00527 |
| GO:0005515 | **receptor binding** | --------- |
| GO:0070696 | transmembrane receptor protein serine/threonine kinase binding | 0.00253 |
| GO:0033612 | receptor serine/threonine kinase binding | 0.00590 |
| GO:0097159 | **organic cyclic compound binding** | --------- |
| GO:1901265 | nucleoside phosphate binding | 0.00527 |
| GO:0005515 | **protein binding** | --------- |
| GO:0070697 | activin receptor binding | 0.00771 |
| GO:0070699 | type II activin receptor binding | 0.00771 |
| GO:0009055 | electron carrier activity | 2.9e-08 |
| GO:0005215 | transporter activity | --------- |
| GO:0022892 | **substrate-specific transporter activity** | --------- |
| GO:0015370 | solute:sodium symporter activity | 1.5e-07 |
| GO:0015294 | solute:cation symporter activity | 1.8e-05 |
| GO:0005343 | organic acid:sodium symporter activity | 0.00013 |
| GO:0017153 | sodium:dicarboxylate symporter activity | 0.00048 |
| GO:0015081 | sodium ion transmembrane transporter activity | 0.00079 |
| GO:0005310 | dicarboxylic acid transmembrane transporter activity | 0.00735 |
| GO:0015501 | glutamate:sodium symporter activity | 0.00771 |
| GO:0005326 | **neurotransmitter transporter activity** | 0.00035 |
| GO:0005328 | neurotransmitter:sodium symporter activity | 0.00035 |
| GO:0022857 | **transmembrane transporter activity** | --------- |
| GO:0015293 | symporter activity | 0.00926 |
| GO:0015296 | anion:cation symporter activity | 0.00994 |
| GO:0060089 | molecular transducer activity | --------- |
| GO:0004871 | **signal transducer activity** | --------- |
| GO:0004872 | receptor activity | 0.00210 |
| GO:0005001 | transmembrane receptor protein tyrosine phosphatase activity | 0.00026 |
| GO:0019198 | transmembrane receptor protein phosphatase activity | 0.00026 |
| GO:0005044 | scavenger receptor activity | 0.00131 |
| GO:0004955 | prostaglandin receptor activity | 0.00253 |
| GO:0004888 | transmembrane signaling receptor activity | 0.00298 |
| GO:0038024 | cargo receptor activity | 0.00320 |
| GO:0004953 | icosanoid receptor activity | 0.00590 |
| GO:0004954 | prostanoid receptor activity | 0.00590 |
| GO:0016209 | antioxidant activity | 0.00075 |
| GO:0045174 | **glutathione dehydrogenase (ascorbate) activity** | 0.00771 |
| GO:0005198 | structural molecule activity | --------- |
| GO:0005201 | **extracellular matrix structural constituent** | 0.00275 |

**Supplementary Table 4. Significantly enriched gene ontologies for cellular components from the low salinity population.**

Significantly enriched gene ontologies in 1:0 asymmetric genes from the low salinity population are ordered by p-value. The p-value is derived from a Fisher’s exact test implemented in topGO from Bioconductor.

| GO ID | Term | p-value |
| --- | --- | --- |
| GO:0016021 | integral to membrane | 0.00037 |
| GO:0031224 | intrinsic to membrane | 0.00058 |
| GO:0000795 | synaptonemal complex | 0.00135 |
| GO:0005887 | integral to plasma membrane | 0.00305 |
| GO:0071944 | cell periphery | 0.00333 |
| GO:0042383 | sarcolemma | 0.00434 |
| GO:0031226 | intrinsic to plasma membrane | 0.00490 |
| GO:0044459 | plasma membrane part | 0.00624 |
| GO:0044425 | membrane part | 0.00663 |
| GO:0000794 | condensed nuclear chromosome | 0.00729 |
| GO:0043025 | neuronal cell body | 0.00802 |
| GO:0005886 | plasma membrane | 0.00815 |

**Supplementary Table 5. Significantly enriched gene ontologies for cellular components from the high salinity population.**

Significantly enriched gene ontologies in 1:0 asymmetric genes from the high salinity population are ordered by p-value. The p-value is derived from a Fisher’s exact test implemented in topGO from Bioconductor.

| GO ID | Term | p-value |
| --- | --- | --- |
| GO:0005576 | extracellular region | 6.4e-08 |
| GO:0031224 | intrinsic to membrane | 6.4e-06 |
| GO:0005886 | plasma membrane | 1.3e-05 |
| GO:0016021 | integral to membrane | 3.1e-05 |
| GO:0071944 | cell periphery | 3.1e-05 |
| GO:0044425 | membrane part | 0.00013 |
| GO:0016020 | membrane | 0.00055 |
| GO:0031012 | extracellular matrix | 0.00061 |
| GO:0005578 | proteinaceous extracellular matrix | 0.00082 |
| GO:0044421 | extracellular region part | 0.00182 |
| GO:0097060 | synaptic membrane | 0.00430 |
| GO:0005615 | extracellular space | 0.00450 |
| GO:0032992 | protein-carbohydrate complex | 0.00532 |
| GO:0071666 | Slit-Robo signaling complex | 0.00532 |

**Supplementary Table 6. Significantly enriched gene ontologies for molecular function from the low salinity population.**

Significantly enriched gene ontologies in 1:0 asymmetric genes from the low salinity population are ordered by p-value. The p-value is derived from a Fisher’s exact test implemented in topGO from Bioconductor.

| GOID | Term | p-value |
| --- | --- | --- |
| GO:0034061 | DNA polymerase activity | 1.7e-21 |
| GO:0003964 | RNA-directed DNA polymerase activity | 5.9e-18 |
| GO:0016779 | nucleotidyltransferase activity | 1.2e-16 |
| GO:0004518 | nuclease activity | 3.2e-13 |
| GO:0003676 | nucleic acid binding | 2.9e-10 |
| GO:0004519 | endonuclease activity | 8.4e-09 |
| GO:0003887 | DNA-directed DNA polymerase activity | 6.4e-07 |
| GO:0004930 | G-protein coupled receptor activity | 6.6e-07 |
| GO:0004190 | aspartic-type endopeptidase activity | 1.0e-06 |
| GO:0070001 | aspartic-type peptidase activity | 2.2e-06 |
| GO:0016772 | transferase activity, transferring phosphate-containing groups | 8.5e-06 |
| GO:0016788 | hydrolase activity, acting on ester bonds | 8.2e-05 |
| GO:0038023 | signaling receptor activity | 0.00010 |
| GO:0004872 | receptor activity | 0.00011 |
| GO:0004888 | transmembrane signaling receptor activity | 0.00014 |
| GO:0003677 | DNA binding | 0.00014 |
| GO:0016787 | hydrolase activity | 0.00048 |
| GO:0000405 | bubble DNA binding | 0.00049 |
| GO:0004386 | helicase activity | 0.00152 |
| GO:0004871 | signal transducer activity | 0.00200 |
| GO:0060089 | molecular transducer activity | 0.00200 |
| GO:1901363 | heterocyclic compound binding | 0.00208 |
| GO:0097159 | organic cyclic compound binding | 0.00254 |
| GO:0004527 | exonuclease activity | 0.00287 |
| GO:0046914 | transition metal ion binding | 0.00332 |
| GO:0008270 | zinc ion binding | 0.00349 |
| GO:0004854 | xanthine dehydrogenase activity | 0.00431 |
| GO:0004855 | xanthine oxidase activity | 0.00431 |
| GO:0016726 | oxidoreductase activity, acting on CH or CH2 groups, NAD or NADP as acceptor | 0.00431 |
| GO:0016727 | oxidoreductase activity: acting on CH or CH2 groups, oxygen as acceptor | 0.00431 |
| GO:0016725 | oxidoreductase activity: acting on CH or CH2 groups | 0.00460 |
| GO:0008265 | Mo-molybdopterin cofactor sulfurase activity | 0.00619 |
| GO:0009378 | four-way junction helicase activity | 0.00619 |
| GO:0035312 | 5'-3' exodeoxyribonuclease activity | 0.00619 |
| GO:0042302 | structural constituent of cuticle | 0.00619 |
| GO:0043176 | amine binding | 0.00619 |
| GO:0045145 | single-stranded DNA specific 5'-3' exodeoxyribonuclease activity | 0.00619 |
| GO:0046873 | metal ion transmembrane transporter activity | 0.00666 |
| GO:0005262 | calcium channel activity | 0.00867 |

**Supplementary Table 7. Significantly enriched gene ontologies for molecular function from the high salinity population.**

Significantly enriched gene ontologies in 1:0 asymmetric genes from the high salinity population are ordered by p-value. The p-value is derived from a Fisher’s exact test implemented in topGO from Bioconductor.

| GO ID | Term | p-value |
| --- | --- | --- |
| GO:0003964 | RNA-directed DNA polymerase activity | 1.5e-07 |
| GO:0034061 | DNA polymerase activity | 1.2e-06 |
| GO:0004872 | receptor activity | 7.8e-06 |
| GO:0016779 | nucleotidyltransferase activity | 7.1e-05 |
| GO:0022836 | gated channel activity | 0.00022 |
| GO:0022839 | ion gated channel activity | 0.00022 |
| GO:0038023 | signaling receptor activity | 0.00036 |
| GO:0004888 | transmembrane signaling receptor activity | 0.00040 |
| GO:0015276 | ligand-gated ion channel activity | 0.00042 |
| GO:0022834 | ligand-gated channel activity | 0.00042 |
| GO:0008270 | zinc ion binding | 0.00112 |
| GO:0005230 | extracellular ligand-gated ion channel activity | 0.00148 |
| GO:0004930 | G-protein coupled receptor activity | 0.00178 |
| GO:0005216 | ion channel activity | 0.00248 |
| GO:0022838 | substrate-specific channel activity | 0.00311 |
| GO:0015267 | channel activity | 0.00444 |
| GO:0022803 | passive transmembrane transporter activity | 0.00444 |
| GO:0001786 | phosphatidylserine binding | 0.00525 |
| GO:0005231 | excitatory extracellular ligand-gated ion channel activity | 0.00615 |
| GO:0004970 | ionotropic glutamate receptor activity | 0.00618 |
| GO:0046914 | transition metal ion binding | 0.00696 |
| GO:0004890 | GABA-A receptor activity | 0.00710 |
| GO:0005234 | extracellular-glutamate-gated ion channel activity | 0.00837 |
